# *Tropheryma whipplei* bivalvular endocarditis and polyarthralgia: a case report

**DOI:** 10.1186/s13256-015-0746-x

**Published:** 2015-11-18

**Authors:** Janina Rivas Gruber, Rossella Sarro, Julie Delaloye, Jean-Francois Surmely, Giuseppe Siniscalchi, Piergiorgio Tozzi, Cyril Jaques, Katia Jaton, Alain Delabays, Gilbert Greub, Tobias Rutz

**Affiliations:** 1 Department of Internal Medicine, Centre Hospitalier Universitaire Vaudois, Rue du Bugnon 11, 1011 Lausanne, Switzerland; 2 Institute of Pathology, Centre Hospitalier Universitaire Vaudois (CHUV), Rue de Bugnon 25, 1011 Lausanne, Switzerland; 3 Infectious Disease Service, Centre Hospitalier Universitaire Vaudois (CHUV), Rue de Bugnon 48, 1011 Lausanne, Switzerland; 4 Cabinet de Cardiologie, Rue des Charpentiers 9, 1110 Morges, Switzerland; 5 Service of Cardiac Surgery, Centre Hospitalier Universitaire Vaudois (CHUV), Rue de Bugnon 46, 1011 Lausanne, Switzerland; 6 Cabinet Medical, Avenue de Florimont 8, 1006 Morges, Switzerland; 7 Institute of Microbiology, University of Lausanne, Rue de Bugnon 48, 1011 Lausanne, Switzerland; 8 Service of Cardiology, Centre Hospitalier Universitaire Vaudois (CHUV), Rue du Bugnon 46, 1011 Lausanne, Switzerland

**Keywords:** Blood culture-negative endocarditis, *Tropheryma whipplei*, Arthralgia

## Abstract

**Introduction:**

*Tropheryma whipplei* infection should be considered in patients with suspected infective endocarditis with negative blood cultures. The case (i) shows how previous symptoms can contribute to the diagnosis of this illness, and (ii) elucidates current recommended diagnostic and therapeutic approaches to Whipple's disease.

**Case presentation:**

A 71-year-old Swiss man with a past history of 2 years of diffuse arthralgia was admitted for a possible endocarditis with severe aortic and mitral regurgitation. Serial blood cultures were negative. Our patient underwent replacement of his aortic and mitral valve by biological prostheses. *T. whipplei* was documented by polymerase chain reactions on both removed valves and on stools, as well as by valve histology. A combination of hydroxychloroquine and doxycycline was initiated as lifetime treatment followed by the complete disappearance of his arthralgia.

**Conclusions:**

This case report underlines the importance of considering *T. whipplei* as a possible causal etiology of blood culture-negative endocarditis. Lifelong antibiotic treatment should be considered for this pathogen (i) due to the significant rate of relapses, and (ii) to the risk of reinfection with another strain since these patients likely have some genetic predisposition.

## Introduction

Blood culture-negative endocarditis (BCNE) represents an important challenge for diagnosis and is therefore associated with higher morbidity and mortality than blood culture-positive endocarditis [1]. BCNE is defined as definite or probable endocarditis according to Duke’s criteria with at least three aerobic and anaerobic blood cultures remaining negative despite prolonged incubation [1]. BCNE represents about 3 % of all endocarditis cases. The most common cause is prior administration of antibiotics followed by fastidious bacteria such as *Coxiella burnetti*, *Bartonella* species and *Tropheryma whipplei*. Incidence of BCNE of unexplained etiology is decreasing thanks to improved diagnostic techniques, including eubacterial PCRs, improved culture-based approaches, as well as species-specific PCRs, serology and immunohistology [2]. In this case report we describe a patient with a history of 2 years of migratory joint pain who presented with dyspnea due to endocarditis finally attributed to *T. whipplei*.

## Case presentation

**Fig. 1:**
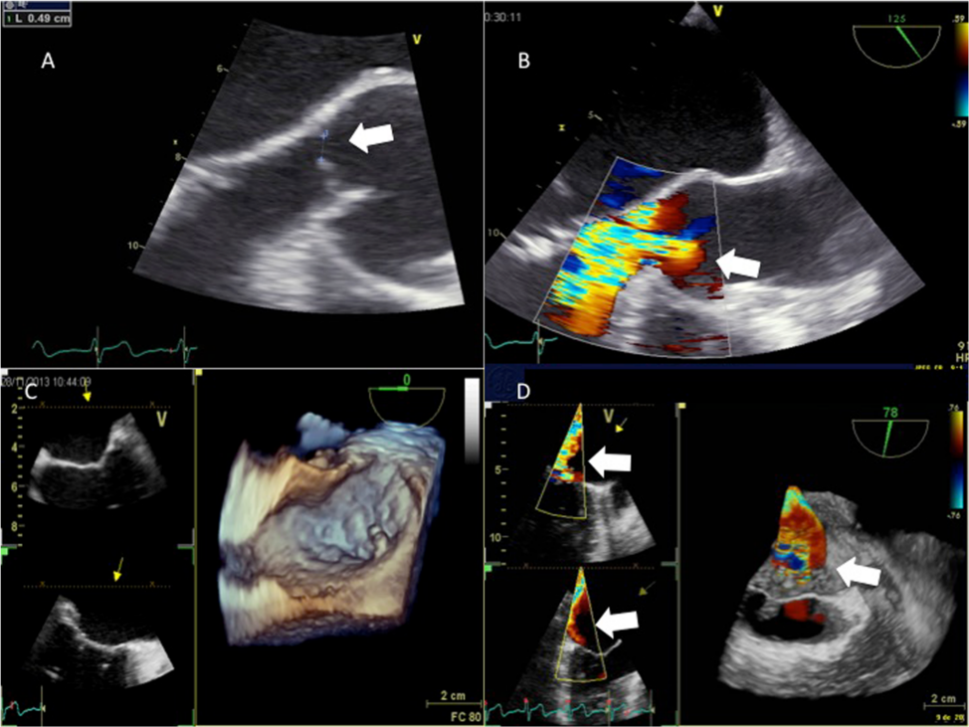
Transoesophageal echocardiographic views of the aortic and mitral valve. **a** Visualization of the perforation of aortic cusp (*arrow*). **b** Visualization of the large regurgitant jet through perforated aortic cusp (*arrow*). **c** Three-dimensional transoesophageal echocardiography of mitral valve. **d** Three-dimensional transoesophageal echocardiography: visualization of regurgitant color Doppler jet through mitral valve (*arrows*)

**Fig. 2:**
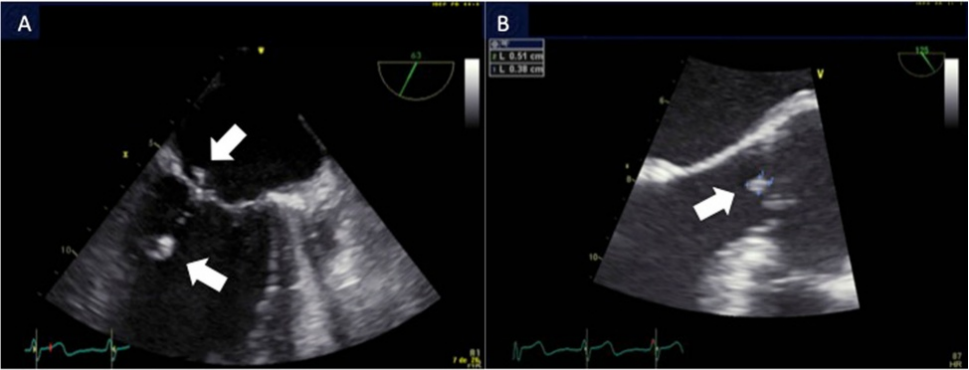
Transoesophageal echocardiographic views of the aortic and mitral valve. **a** Visualization of subacute vegetations on posterior leaflet and chordae tendinae of mitral valve (*arrows*). **b** Visualization of a vegetation on aortic valve (*arrow*)

A 71-year-old Caucasian man, known as a HLA B27 carrier complained of progressive dyspnea for 4 months without any other respiratory or cardiovascular symptom. His medical history revealed a metabolic syndrome and migratory joint pains for the past 2 years without signs of local inflammation. Extensive investigations failed to identify a precise diagnosis and nonsteroidal anti-inflammatory drugs (NSAIDs) were empirically prescribed. He was not known for past history of cardiopulmonary disease and the rest of his past medical history was noncontributory. On physical examination, systolic and diastolic heart murmurs were heard at the precordium. An outpatient transthoracic echocardiography revealed aortic and mitral regurgitation and vegetations of both valves were suspected. Our patient was admitted to a regional hospital. On admission, he complained of dyspnea at rest (New York Heart Association Functional Classification class IV) and orthopnea and reported a single episode of fever (38.8 °C) without chills a week before. On clinical examination, our patient presented signs of acute heart failure with bilateral crackles on pulmonary auscultation, elevated jugular venous pressure and peripheral edema. Cardiac auscultation revealed a 3/6 decrescendo diastolic murmur over the aortic and a 2/6 holosystolic murmur over the mitral region. The routine laboratory workup was unremarkable with a white blood cell count of 4.1 g/L, and a C-reactive protein level of 21 mg/L (normal value <10 mg/L). Four pairs of blood cultures were drawn, which remained negative. Transesophageal echocardiography showed severe aortic regurgitation due to left coronary leaflet perforation (Fig. 1a and b), significant mitral regurgitation due to rupture of the chordae tendineae (Fig. 1c and d), and the presence of vegetations on both valves (size <15 mm) (Fig. 2a and b). The left ventricle (LV) was hypertrophied and enlarged with mild systolic dysfunction (LV ejection fraction 53 %). A computed tomography scan of his brain was negative for distant septic emboli. Our patient was admitted to the intensive care unit with the diagnosis of a possible endocarditis with severe bivalvular regurgitation. Empirical antibiotic therapy was initiated with amoxicillin-clavulanate and gentamicin. In addition, intravenous furosemide and oxygen were administered to treat heart failure. As blood cultures showed no growth after 14 days of incubation including for HACEK (*Haemophilus* species, *Aggregatibacter* species, *Cardiobacterium hominis*, *Eikenella corrodens*, and *Kingella* species) bacteria, a search for fastidious bacteria by serology and polymerase chain reaction (PCR) was performed. Moreover, the antibiotic spectrum was widened by the addition of ciprofloxacin and doxycycline.

**Fig. 3:**
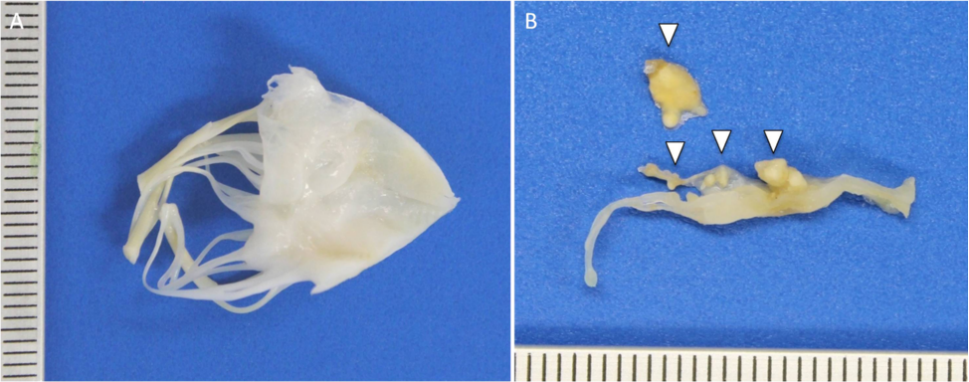
Histopathology findings: gross appearance of *T. whipplei-*infected native mitral valve specimen. **a** Unremarkable atrial side of valvular leaflet. **b** Transverse section of valve harboring voluminous bright yellow vegetations (*arrowheads*), up to 6 mm exclusively confined to the ventricular side of the valve and chordae tendinae

**Fig. 4:**
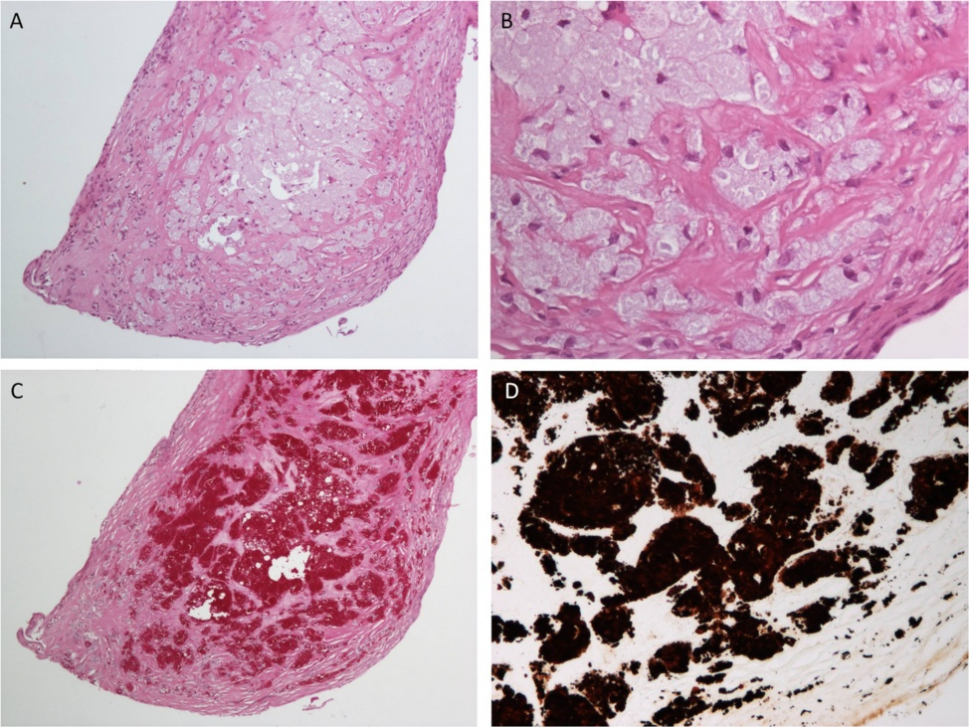
Histopathology findings: microscopic appearance of *T. whipplei-*infected native mitral valve specimen. **a** On low-power view, dense foamy macrophage infiltrate within the valve (hematoxylin and eosin, original magnification × 200). **b** On high-power view, macrophages with light gray and granular cytoplasm (hematoxylin and eosin, original magnification × 400). **c** The cytoplasm is filled with periodic acid-Schiff-positive material (original magnification × 200). **d** The cytoplasmatic periodic acid-Schiff-positive material is also strongly positive with Warthin-Starry stain (original magnification × 400)

The patient was transferred to our hospital for further treatment. Coronary angiography was normal. Our patient underwent successful replacement of the aortic and mitral valves by biological prostheses with an uneventful recovery. Gross examination of the valves revealed multiple yellowish vegetations on the ventricular side of the mitral (Fig. 3) and aortic valve. Histology showed confluent areas of foamy macrophages on hematoxylin and eosin (Fig. 4a and b), strongly colored by periodic acid-Schiff (PAS) and Warthin-Starry staining (Fig. 4c and d, respectively). The rest of the valves showed minor degenerative changes in the form of light fibrosis, myxoid stroma and focal calcifications, without neovascularization. Both valves were positive for *T. whipplei* with specific PCRs adapted from Fennolar *et al.* with 18,000,000 copies/mL on the aortic valve [3]. In addition, the same *T. whipplei-*specific PCR of the stools was positive with 32,000 copies/mL; however, the *T. whipplei* PCRs of saliva and blood returned negative. No upper gastrointestinal investigations were performed given the absence of symptoms. PCR and serology results for *Coxiella burnetti* and *Bartonella henselae* were negative. The antibiotic treatment was switched to ceftriaxone and gentamicin for 2 weeks and then relayed by doxycycline and hydroxychloroquine orally for lifetime.

At his 3-month follow-up, our patient had no cardiac complaints except a mild dyspnea at exercise. Echocardiography confirmed a good function of both prostheses without leakage but persistence of a systolic dysfunction (LV ejection fraction 45 %). Interestingly, our patient reported the complete disappearance of his arthralgia since the introduction of the antibiotic treatment.

## Discussion

Classical Whipple’s disease (cWD) is a late-onset chronic multisystemic infectious disease caused by *T. whipplei* [4, 5]. The transmission of the pathogen is probably related to the fecal-oral route [6]. The classical clinical manifestations of cWD are weight loss, diarrhea, abdominal pain and polyarthralgia [5]. The extraintestinal manifestations of cWD are most commonly cardiac and neurologic, but osteoarticular infections, uveitis, mucocutaneous and pulmonary diseases have also been described [5, 7–9]. It is mainly a disease of middle-aged white men and it is suspected that an immunogenetic susceptibility might be required for developing the late-onset disease [6]. Our patient is a HLA B27 carrier; an association of cWD and HLA B27 has been described by Feurle *et al.* in 1979 [10].

Whipple’s endocarditis is a frequent chronic extraintestinal manifestation due to *T. whipplei* infection and it represents one of the most common causes of BCNE [6]. A high prevalence of Whipple’s disease has been reported in eastern-central France, Switzerland and Germany. It is not known if it is due to the high prevalence of the agent in this area or whether it is a result of a better search for the disease [11, 12]. Geissdörfer *et al.* found in a cohort study in Germany that *T. whipplei* was the fourth most frequent pathogen (6.3 %) of 255 cases of bacterial endocarditis after streptococci, staphylococci and enterococci listed as the main pathogens [13]. As it is for cWD, Whipple’s endocarditis affects mainly white men in their sixties [8, 11]. The clinical presentation is primarily due to the valvular lesions manifested by acute heart failure. Arthralgia or arthritis often precedes the diagnosis of endocarditis as shown by Fenollar *et al.* in their observational cohort study of 28 cases of Whipple’s endocarditis. In this study, 75 % of patients reported previous joint pain [11]. Fever, however, is described in only 25–30 % of cases and gastrointestinal symptoms appeared to be rare [6, 11]. Thus, the Duke criteria that are proposed for the diagnosis of endocarditis are usually not helpful in this context. Our patient also presented mainly with cardiovascular symptoms and a single episode of fever. Moreover, and typical for Whipple’s endocarditis, he presented with arthralgia for 2 years, for which no etiology was identified. Interestingly, the arthralgia disappeared with the antibiotic treatment. Therefore, arthralgia was an important diagnostic clue in his medical history and should always be looked for in BCNE. Previous heart valve disease has been described in about one third of cases, but was not present in our patient. Aortic and mitral valves are most frequently involved with a few reports of tricuspid endocarditis [6, 11, 14].

The diagnosis of Whipple’s endocarditis is mainly confirmed by surgically obtained specimens of the involved cardiac valves. The pathologic features of the valves include vegetations of different size, significant fibrosis and the presence of foamy macrophages containing numerous PAS-positive inclusions with slight inflammation [2, 8, 13]. However, PAS staining is not specific for *T. whipplei* infection since PAS-positive macrophages have been found with other infectious agents [8]. Currently, molecular analysis of the valves using a specific PCR is the best diagnostic approach [6, 15]. Although screening of saliva and fecal specimens by PCR has a high negative predictive value in diagnosing cWD [6], it is not the case for Whipple’s endocarditis. In addition, PCR assays of blood samples showed a low sensitivity for the disease [6, 11]. On the other hand, growth of *T. whipplei* cannot be really considered as a diagnostic tool due to the difficulty of the technique and the very few specialized laboratories available. Improvement of diagnostic tools is therefore necessary to allow a better diagnosis of Whipple’s endocarditis before cardiac surgery.

Regarding the treatment of Whipple’s endocarditis, there is no standard antibiotic strategy and it is generally based on the therapeutic protocol for cWD. Prior cases have reported failure of treatment with trimethoprim-sulfametoxazole in cWD and also in Whipple’s endocarditis [16, 17]. Moreover, trimethoprim is not active on *T. whipplei* since its drug target is missing. Thus, some authors recommend 2 weeks of ceftriaxone followed by a combination of doxycycline and hydroxychloroquine with control of drug concentration in the blood every 3 months [8, 11]. The length of the treatment has not been defined precisely, and an antibiotic course of 18–24 months is often proposed in the literature. However, some experts recommend a lifelong treatment considering that a genetic susceptibility of the host likely plays a role in relapses and reinfection with another strain [18]. Such a lifelong treatment was prescribed to our patient. Our patient was informed of the risk of photosensitivity and retinal toxicity related to doxycycline and hydroxychloroquine, respectively. Rarely, hydroxychloroquine can induce a toxic cardiomyopathy manifested by, for example, severe systolic dysfunction [19]. Follow-up includes control of the plasma level of antibiotics, blood count and hepatic and renal function and a regular ophthalmological control. In addition, PCR on saliva and stools may be done 3 and 6 months after treatment start, and then once a year to possibly detect early relapse or reinfection.

## Conclusions

We report a typically subacute presentation of infective endocarditis due to *T. whipplei* with a previous history of migrant arthralgia of unknown etiology. This case highlights the importance of looking for unusual fastidious bacteria in the occurrence of BCNE endocarditis as soon as blood cultures return with negative results. Investigations should systematically include *T. whipplei* given its common occurrence in such settings. Lifelong antibiotic treatment could be considered to treat *T. whipplei* endocarditis even if prospective studies are lacking.

## Consent

Written informed consent was obtained from the patient for publication of this case and any accompanying images. A copy of the written consent is available for review by the Editor-in-Chief of this journal.

## Abbreviations

BCNE: blood culture-negative endocarditis
cWD: classical Whipple’s disease
LV: left ventricle
NSAIDs: nonsteroidal anti-inflammatory drugs
PAS: periodic acid-Schiff stain
PCR: polymerase chain reaction
